# Lateral Gradient Ambidextrous Optical Reflection in Self-Organized Left-Handed Chiral Nematic Cellulose Nanocrystals Films

**DOI:** 10.3389/fbioe.2021.608965

**Published:** 2021-02-05

**Authors:** Jiawei Tao, Jiaqi Li, Xiao Yu, Lihong Wei, Yan Xu

**Affiliations:** State Key Laboratory of Inorganic Synthesis and Preparative Chemistry, Jilin University, Changchun, China

**Keywords:** cellulose nanocrystals, tilted-angle self-assembly, gradient photonic bandgaps, circularly polarized light, ambidextrous reflection

## Abstract

Artificial photonic materials displaying ordered reflected color patterns are desirable in the field of photonic technologies, however, it is challenging to realize. Here we present that self-assembly of cellulose nanocrystals (CNC) in a tilted cuvette leads to the formation of rainbow color CNC films. We show that the self-organized CNC films enable simultaneous reflection of left-handed circularly polarized (LCP) and right-handed circularly polarized (RCP) light with lateral gradient transmittance ratio (LCP/RCP: 8.7–0.9) and the maximum reflectance value up to *ca.* 72%. This unique ambidextrous optical reflection arises from left-handed chiral photonic architectures with lateral gradient photonic bandgaps and nematic-like defects at the film-substrate interface and between left-handed photonic bandgap layers acting as a half-wavelength retarder. We demonstrate that the tilted angle self-assembly method provides a feasible step toward color patterning of CNC-based photonic films capable of ambidextrous optical reflection.

## Introduction

Photonic crystals possessing periodic modulation of dielectric constants and photonic bandgaps (PBG) offer ways to manipulate light, which are of significance for applications including circular polarizers (Lv et al., 2019), photonic devices (Tao et al., 2020a), chiral catalysts (Cho et al., 2016), and biosensors (Lv et al., 2017). Chiral nematic materials are attractive as self-assembled one-dimensional photonic crystals displaying unique circular polarization ability. Structural coloration arising from light-matter interactions is common among many living organisms in nature (Zhao et al., 2012; Almeida et al., 2018). For example, helicoidal nanostructures of cellulose nanofibrils are responsible for the intense blue color found in many fruits and leaves (Vignolini et al., 2012). Similar phenomenon has been observed in some beetle cuticles. Of particular interests are the cuticle of beetle *Plusiotis resplendens* displaying optically ambidextrous reflection in a left-handed chiral nematic organization and the cuticle of beetle *Chrysina gloriosa* enabling strong polarization-insensitive reflection (Sharma et al., 2009). This effect originates from a nematic-like layer, which is sandwiched between two left-handed PBG layers, acting as a half-wavelength retarder (Caveney, 1971). These structure-endowed chiroptical activities have inspired the design and fabrication of artificial chiral photonic materials. In particular, cellulose nanocrystals (CNC) derived from natural cellulose self-assemble in colloidal dispersions into thermodynamically more stable left-handed helicoids that can be preserved upon drying. Evaporation-induced self-assembly on a planar surface is the common method used to fabricate photonic CNC films (Lagerwall et al., 2014; Tran et al., 2018; Tao and Xu, 2020). The left-handed chiral nematic structure of CNC has intrinsic ability to enable photonic bandgap-based reflection of left-handed circularly polarized (LCP) light, transmission of right-handed circularly polarized (RCP) light and RCP spontaneous luminescence (Klemm et al., 2011; Zheng et al., 2018; Tran et al., 2020). The wavelength of selective reflection follows the Eq. 1,

(1) $$\lambda \,\left(\theta \right)=n_{a\,v\,g}\,P\,s\,i\,n\,\theta ,$$

where *n*_*avg*_ is the average refractive index, *P* is the helical pitch and θ is the incident angle from nematic surface (Parker et al., 2018). Leveraging on CNC self-assembly thermodynamics vs. kinetics (Wang et al., 2016), quasinematic structures (Hiratani et al., 2017), nematic structures (Gan et al., 2019), microgap-embedded chiral photonic architectures (Fernandes et al., 2017), surface-textured chiral photonic architectures (Tran et al., 2018), and chiral nematic-nematic photonic architectures have been spontaneously organized, manifesting the wealth of CNC photonic materials (Tao et al., 2020b). Self-assembly of CNC in magnetic and electric fields narrow helical pitch and orientation polydispersity, which may facilitate the development of high efficiency circularly polarized luminescence materials (Frka-Petesic et al., 2017a,b). So far, chiral photonic CNC films with continuously varying PBG, to the best of our knowledge, has not been reported.

Here we report that self-assembly of CNC in a titled cuvette of stable colloidal dispersions produces chiral photonic CNC films that display rainbow colors and lateral gradient ambidextrous optical reflection. The CNC film features a photonic architecture formed with left-handed PBG layers and nematic-like layers at the film-substrate interface and embedded between PBG layers. It enables ambidextrous optical reflection showing lateral gradient transmittance values from the higher to the lower end of the cuvette. The ambidextrous chiroptical properties are owing to the half-wavelength retardation of nematic-like phases nucleated *in situ* and kinetically arrested. The transmittance values of the RCP light and LCP light are tilt angle-dependent, which appear most pronounced at 30°. Increasing the tilt angle weakens chiral nematic features and promotes unidirectional alignment of CNC. We demonstrate that tilted-angle self-assembly allows synergistic self-assembly and kinetic stabilization, providing a step toward self-organized chiral nematic-nematic films capable of color patterning, lateral gradient PBG and ambidextrous optical reflectivity.

## Materials and Methods

### Materials

Cotton pulp board was purchased from Hebei Paper Group, China. Sulfuric acid was purchased from Beijing Chemical Company. Dialysis bags were purchased from Spectrum labs with 28 mm in diameter and molecular cut-off of 0.8–15 kDa. Left and right circular polarization filters were purchased from Jinan Photoelectric Technology Company with effective spectral range of 400–700 nm. All reagents were used as received without further purification.

### Preparation of CNC Suspension

CNC was obtained from cotton pulp by acid hydrolysis using sulfuric acid (64%) and the acid/cotton pulp mass ratio was kept at 10:1. The hydrolysis reaction took place at 45°C for 90 min under vigorous stirring, and the reaction was terminated using deionized water. The solid product was centrifuged (8,000 r/min, 6 min) several times and dialyzed against deionized water until pH = 7. The suspension was diluted to desired concentrations for keeping.

### Preparation of CNC-α Film

**FIGURE 1:**
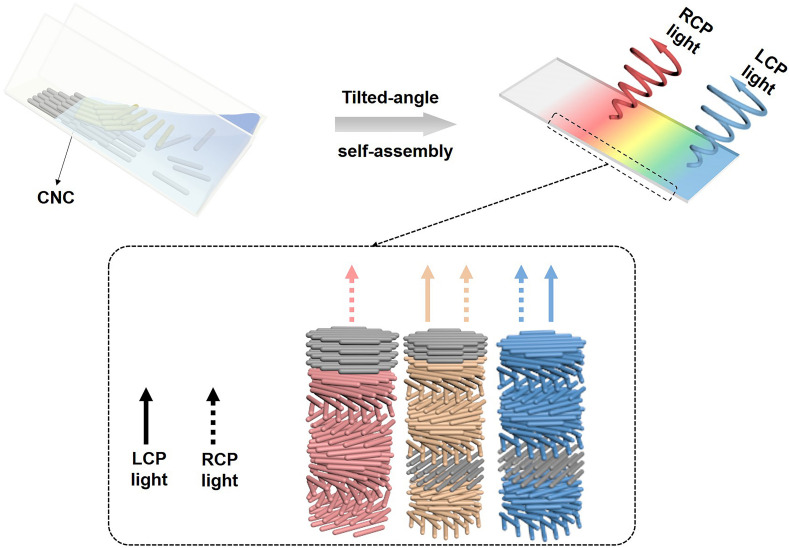
Tilted-angle self-assembly leads to self-organized CNC films displaying rainbow colors and ambidextrous optical reflection. LCP light arises from selective reflection of incident light by left-handed chiral nematic structure. RCP light is due to *in situ* reversal of transmitted RCP light by embedded nematic-like layers that are found at the film-substrate interface as well as embedded between left-handed PBG layers. The nematic-like layer at the film-substrate interface above left-handed PBG layer causes *in situ* handedness reversal of LCP light to RCP light; the nematic-like layer embedded between two same left-handed PBG layers converts transmitted RCP light to LCP light, then reflected by the left-handed PBG layer below and converted further to RCP light as it propagates back through the nematic-like layer. Such mesostructural feature causes a total reflectance exceeding the 50% limit of a left-handed chiral nematic structure. Note: gray for nematic-like order; solid arrows for LCP light; dotted arrow for RCP light.

Two milliliter of CNC suspensions (4 wt%, pH = 7) without macroscopic phase separation was allowed to assemble in a tilted quartz cuvette (25°C) as illustrated in Figure 1. Changing the tilt angle (α) of the cuvette to base plane led to a range of iridescent rainbow color CNC films, denoted as CNC-α.

## Results and Discussion

**FIGURE 2:**
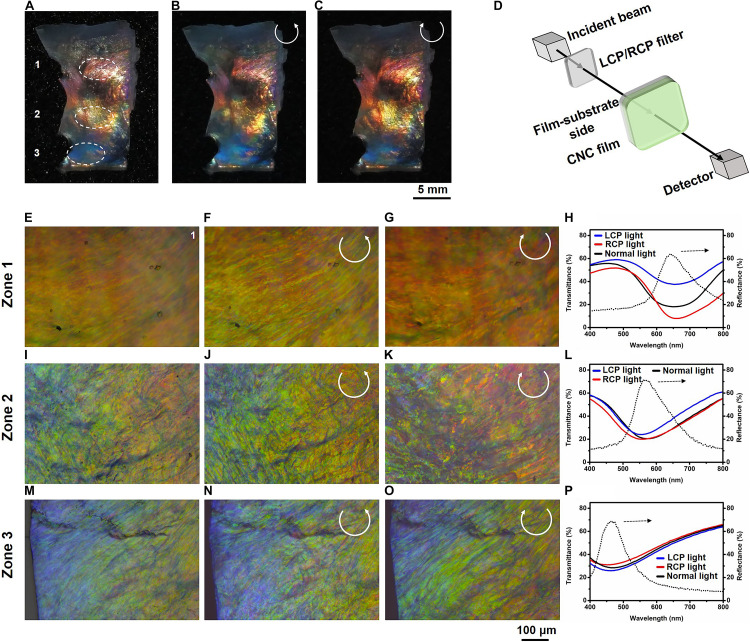
**(A–C)** Photographs of CNC-30 viewed under normal light **(A)**, through the LCP filter **(B)** and through the RCP filter **(C)** showing lateral gradient color pattern. **(D)** Schematics illustrating the spectral measurement by exposing the film-substrate side to incident beam. Reflected optical micrographs of the zones 1, 2, 3 viewed under normal light **(E,I,M)**, through the LCP filter **(F,J,N),** and the RCP filter **(G,K,O)**, respectively. **(H,L,P)** The transmission spectra (left vertical axis) of the zones 1, 2 and 3 probed by normal light (solid black lines), through the LCP filter (solid blue lines) and the RCP filter (solid red lines) and reflection spectra (right vertical axis) probed by normal light (dotted black lines).

CNC-30 film showed colors in order of red, orange, green and blue from the higher to the lower end of the cuvette against a black background when viewed in normal white light (Figure 2A). Notably, the portion of the film at the air-substrate interface at the higher end was colorless. The film colors dimmed when viewed through a 400–700 nm left-handed circular polarization (LCP) filter and intensified when viewed through a 400–700 nm right-handed circular polarization (RCP) filter (Figures 2B,C). It indicates that CNC-30 reflects simultaneously LCP and RCP light.

The self-assembly of CNC in the titled cuvette of stable dispersion experiences variation in kinetic factors including CNC concentration, assembly time and interfacial interactions that may lead to continuous change in the photonic properties. The CNC-30 film was laterally segmented into zones 1, 2, and 3 and individually examined using optical microscopy and UV-vis transmission spectroscopy. All spectra were recorded by facing the film-substrate side of films to incident beam (Figure 2D). The green-red reflection pattern of the zone 1 recorded in normal white light appeared a green-red stripped pattern or an intensified green-red pattern with a contrast variation when recorded through the LCP or RCP filter (Figures 2E–G). The corresponding transmission spectra showed simultaneous reflection of LCP and RCP light with the peak transmittance values of *ca.* 38 and 8% at 655 and 660 nm, respectively, consistent with the optical microscopy studies (Figure 2H, left vertical axis). The optical micrographs of the zone 2 displayed a blue-green pattern with a red tint under normal white light that appeared intensified or a green-red pattern with random blue and dark patches when recorded through the LCP or RCP filter (Figures 2I–K). The corresponding transmission spectra revealed simultaneous reflection of LCP and RCP light with the peak transmittance values of *ca.* 24 and 21% at 554 and 562 nm, respectively (Figure 2L, left vertical axis). The zone 3 displayed similar blue and yellow patterns with variations in color contrast recorded in normal white light or through the LCP or RCP filter (Figures 2M–O). The corresponding transmission spectra probed by the LCP or RCP filter revealed LCP or RCP light reflection band with the peak transmittance values of ca. 23 or 31% at 460 or 463 nm (Figure 2P, left vertical axis). We also examined zones 1, 2, 3 using circular dichroism (CD) spectroscopy (Supplementary Figure 1), the corresponding signals go from negative to positive, suggesting a change in chirality. Noteworthy are the ambidextrous optical reflections of CNC-30 with coincided band wavelengths of RCP and LCP light reflection and, more strikingly, a continuous change in the peak wavelength from the zone 1 to the zone 3. The reflection bands probed by normal white light coincided with the corresponding transmission bands in the peak wavelength and showed the peak reflectance values of ca. 64, 72, and 69%, exceeding exclusively the 50% limit of a single-handed chiral nematic phase (Figures 2H,L,P, right vertical axis; Michel and Dessaud, 2006). These results indicated that CNC-30 film was capable of reflecting circularly polarized light of both handedness with lateral gradient colors and chiral distribution.

**FIGURE 3:**
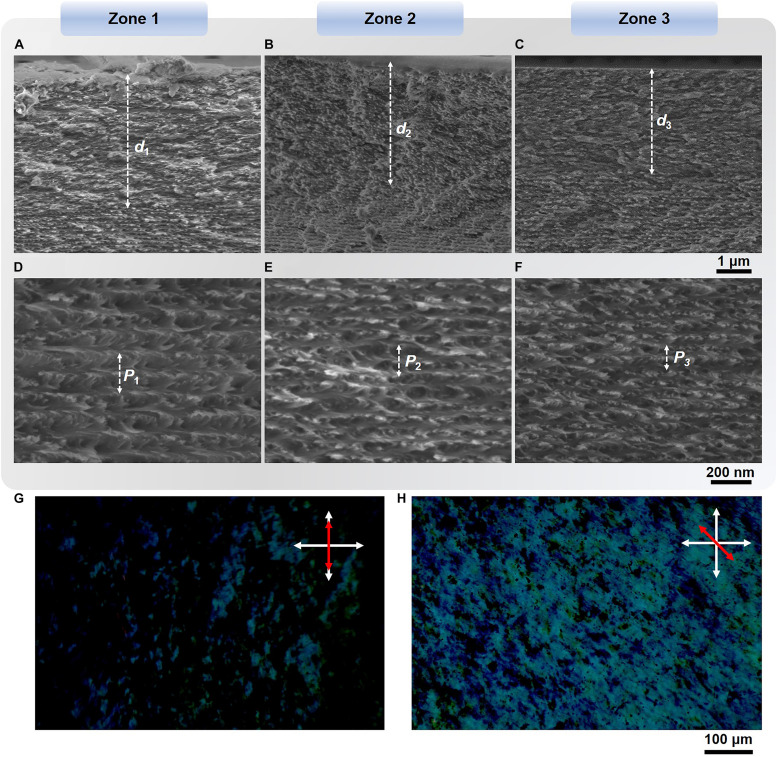
**(A–F)** Cross-sectional SEM images of zones 1, 2, and 3 on CNC-30 show nematic-like layers at the film-substrate interface with the thickness of *ca*. *d*_1_ = 3,938 nm, *d*_2_ = 3,521 nm, and *d*_3_ = 2,971 nm **(A–C)**. High magnification SEM images exhibit left-handed PBG layers with the helical pitches of *P*_1_ = 202 nm, *P*_2_ = 170 nm, and *P*_3_ = 142 nm **(D–F)**. **(G,H)** Transmission polarized optical micrographs of CNC-60 show birefringent patterns with the transmitted intensity change from minimum **(G)** and to maximum at a 45° rotation **(H)**. The white double-headed arrows refer to cross-polarizers and red double-headed arrows represent the CNC director.

We investigated the mesostructural features of the zones 1, 2, and 3 using scanning electron microscopy (SEM). High-magnification SEM images of the film cross-section revealed a chiral nematic-nematic bilayer architecture (Figures 3A–C), where the layer at the air-film interface contained primarily chiral nematic order with the helical pitches of P1 = 202 nm, P2 = 170 nm, and P3 = 142 nm (Figures 3D–F). The chiral nematic order was confirmed to be left-handed based on the Neville convention, while right-handed helicoids were not found (Majoinen et al., 2012). The helical pitch of the chiral nematic phase decreased from the zone 1 to the zone 3, consistent with the optical microscopy and transmission spectral studies (Figure 2). We attributed the decreasing helical pitches to the lateral increasing of local CNC concentration induced by tilt angle assembly (Dumanli et al., 2014). The layer formed at the film-substrate interface contained primarily unidirectionally aligned CNC with respective thickness of d1 = 3,938 nm, d2 = 3,521 nm and d3 = 2,971 nm. Nematic-like layers were also found between left-handed PBG layers and increased in number from the zone 1 to the zone 3, which resulted from reduction in fluidity and kinetically arrest of CNC.

When rotated around a plane perpendicular to the crossed polarizers in a transmission polarizing optical microscopy, the zone 1 showed birefringent patterns and a change in the transmitted intensity of some color appearances from minimum to maximum at a 45° rotation, confirming the presence of nematic-like phase (Figures 3G,H). These nematic-like layers may introduce a wavelength retardation *L* to the propagating light following the Eq. 2,

(2) $$L=\Delta \,n\,d,\Delta \,\text{n}=n_{e}-n_{o},$$

where Δ*n* is the net birefringence, *n*_*e*_ and *n*_*o*_ are the extraordinary and ordinary refractive indices of birefringent medium, respectively (Cruz et al., 2018), and *d* is the optical path length of the nematic-like layer that can be approximated to be the layer thickness. Significantly, half-wavelength retardation causes handedness reversal of propagating circularly polarized light. Taking the zone 1 for example, given that approximately *d_1_* = 3,938 nm and Δ*n* = 0.08 (Krishnaiyer et al., 1968), transmitted RCP light at 630 nm would be half-wavelength retarded leading to LCP light reflection at the same wavelength. When the wavelength of the converted LCP light matches the PBG of subsequent chiral nematic layers, it is then reflected and undergoes further reversal into reflected RCP light as it propagates back through the nematic-like layer as evidenced by the transmission spectra probed by LCP or RCP light (see Figures 2G,K,O). Hence, these novel optical textures can be attributed to the synergy of left-handed chiral organizations with gradient changing helical pitches and nematic-like phases to light, the former leads to a rainbow pattern while the distribution of the latter results in the lateral gradient ambidextrous optical reflection.

**FIGURE 4:**
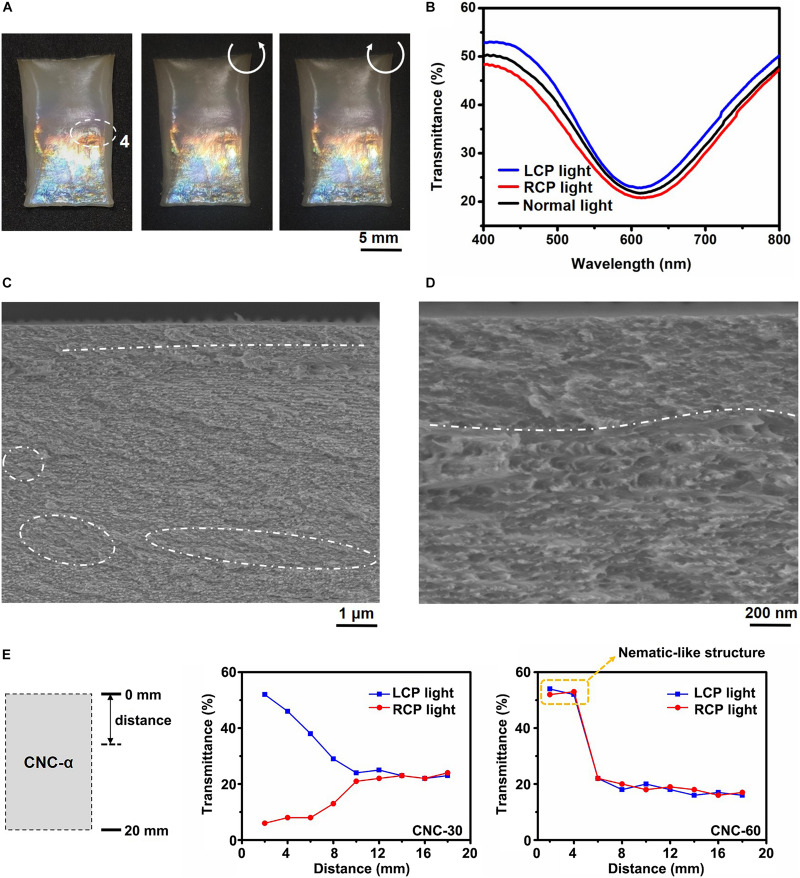
**(A–D)** Photographs of CNC-60 viewed under normal white light (left), through the LCP filter (middle) and the RCP filter (right) **(A)**, the transmission spectra of CNC-60 probed by normal white light (solid black line), through the LCP filter (solid blue line) or the RCP filter (solid red line) **(B)**, SEM images show a chiral nematic-nematic architecture **(C)**, where the nematic-like layers (dotted white circles) are embedded between left-handed PBG layers and at the film-substrate interface (above dotted white lines) **(C,D)**. **(E)** Changes in the transmittance value of the LCP light (blue line) and RCP light (red line) in CNC-30 and CNC-60.

We show that the rainbow color pattern and lateral gradient ambidextrous optical reflection are tilt angle-dependent. Taking CNC-60 for example, the film displayed colors in order of red, orange, yellow and blue under normal white light against a black background, and the color pattern remained literally unchanged when recorded through the LCP filter or RCP filter (Figure 4A). Selective reflection band emerged at the zone 4, next to the non-reflective region on the higher end of films, showed LCP and RCP light reflection bands with the peak transmittance values of 23 or 21% at 617 or 618 nm when probed through the LCP or RCP filter (Figure 4B). Nematic-like phase was found at the film-substrate interface as well as between left-handed PBG layers based on the SEM images (Figures 4C,D, highlighted by dotted white circles, the region above dotted white line). Notably, CNC-60 displayed larger non-reflective region at the cuvette higher end and closely matched transmittance values of the LCP and RCP light reflection compared with CNC-30. In other words, the transmittance ratio of LCP and RCP of CNC-60 was always close to 1.0, while for film CNC-30, the LCP/RCP ratio gradient decreased from 8.7 to 0.9 (Figure 4E).

In order to understand the CNC self-assembly in a tilted cuvette, two control films, CNC-0 and CNC-90, were fabricated in a CNC dispersion (4 wt%, 25°C, pH = 7) by evaporation-induced self-assembly method and by vertical deposition method (Gan et al., 2019), respectively. The CNC-0 film appeared red to the naked eyes under normal white light that intensified or dimmed when viewed through the LCP or RCP filter (Supplementary Figure 2A). One strong LCP or one weak RCP light reflection band (peak transmittance values of 27 and 82% at 644 nm) displayed on the transmission spectrum probed through the LCP or RCP filter (Supplementary Figure 2B). It indicates the presence of embedded nematic-like layer between the left-handed PBG layers, acting as a half-wavelength retarder, as evidenced by the SEM images (Supplementary Figures 2C,D). Vertical immersion of a quartz plate in a colloidal dispersion promotes unidirectional alignment leading to nematic-like CNC film, CNC-90, which is non-reflective in the visible light regime (Supplementary Figure 3). Taken together, CNC favored unidirectional nematic-like phases at the film-substrate interface along the direction of the tilt, which occupied a larger proportion when increasing the tilt angle. At the same time, the thermodynamically stable left-handed chiral nematic phases were weakened.

## Conclusion

We have presented experimental evidences that tilt angle self-assembly produces chiral photonic CNC films displaying rainbow colors and lateral gradient ambidextrous optical reflection that can be tuned by a change in the tilt angle. We demonstrate how the circularly polarized light reflection of both handedness can be manipulated to produce chiral photonic CNC films with changing ambidextrous reflectivity and maximum reflectance values of *ca.* 72%. It allows lateral gradient change in assembly duration and local CNC concentrations and can be manipulated by a change in tilt angle. The formation of nematic-like phase at the film-substrate interface is owing to the combined effect of kinetic stabilization of transient phase and seeded growth. The self-organization of rainbow color CNC films in a tilted cuvette is demonstrated as a step toward synthetic construction of one-dimensional chiral photonic materials displaying optical patterning and ambidextrous reflection with lateral gradient chiroptical properties for photonic applications.

## Data Availability Statement

The original contributions presented in the study are included in the article/Supplementary Material, further inquiries can be directed to the corresponding author/s.

## Author Contributions

JT wrote this manuscript, designed, and carried out the whole experiments. JL and XY contributed to cellulose nanocrystals preparation and partial data collection. LW contributed to SEM imaging collection. YX supervised the work, contributed to data analysis, and manuscript revision. All authors discussed the results and contributed to the completion of the manuscript.

## Conflict of Interest

The authors declare that the research was conducted in the absence of any commercial or financial relationships that could be construed as a potential conflict of interest.
