# Supplementary material for: Lateral Gradient Ambidextrous Optical Reflection in Self-Organized Left-Handed Chiral Nematic Cellulose Nanocrystals Films
Source: Front Bioeng Biotechnol. 2021 Feb 5;9:608965. doi: 10.3389/fbioe.2021.608965 (PMC7892906; doi:10.3389/fbioe.2021.608965)
Supplement: Supplementary file 1 [file Table_1.DOCX]

Supplementary Material

# Supplementary Information

**Characterization**. Electron micrographs were acquired on JEOL-7800F field emission scanning electron microscopy (SEM) at an accelerating voltage of 3.0 kV with a 9.8-10.0 mm working distance. Transmission electron microscope (TEM) imaging was acquired on FEI Tecnai G2S-Twin F20 with a field emission gun operating at 200 kV. UV-Vis transmission spectra were recorded on Analytik Jena Specord 210 plus UV-Vis spectrophotometer probed by normal light, and through left and right circular polarization (LCP, RCP) filters. Reflection spectra were recorded on a Maya 2000 PRO fiber optical spectrometer with Ocean DH-2000-BAL UV-Vis-NIR light source. Circular dichroism spectra were recorded using an Applied Photophysics Chirascan Plus CD spectropolarimeter. Optical micrographs under normal white light were taken on a Leica DM4000M Versatile Upright Microscope in reflection mode. Reflected optical micrographs were acquired under normal white light and by inserting LCP or RCP filters in the light path. Photographs were taken using a Canon EOS 6D under normal white light and 365 nm irradiation. LCP or RCP filters were inserted in front of the camera to collect left and right circular polarization patterns and left- and right-circularly polarized luminescent patterns, respectively. Polarized optical microscopy images and video were taken using a Leica DM4000M Versatile Upright Microscope operating in the reflection mode.

# Supplementary Figures and Tables





**Supplementary Figure S1.** Circular dichroism (CD) spectrum of the zones 1, 2 and 3 of CNC-30 film.

**
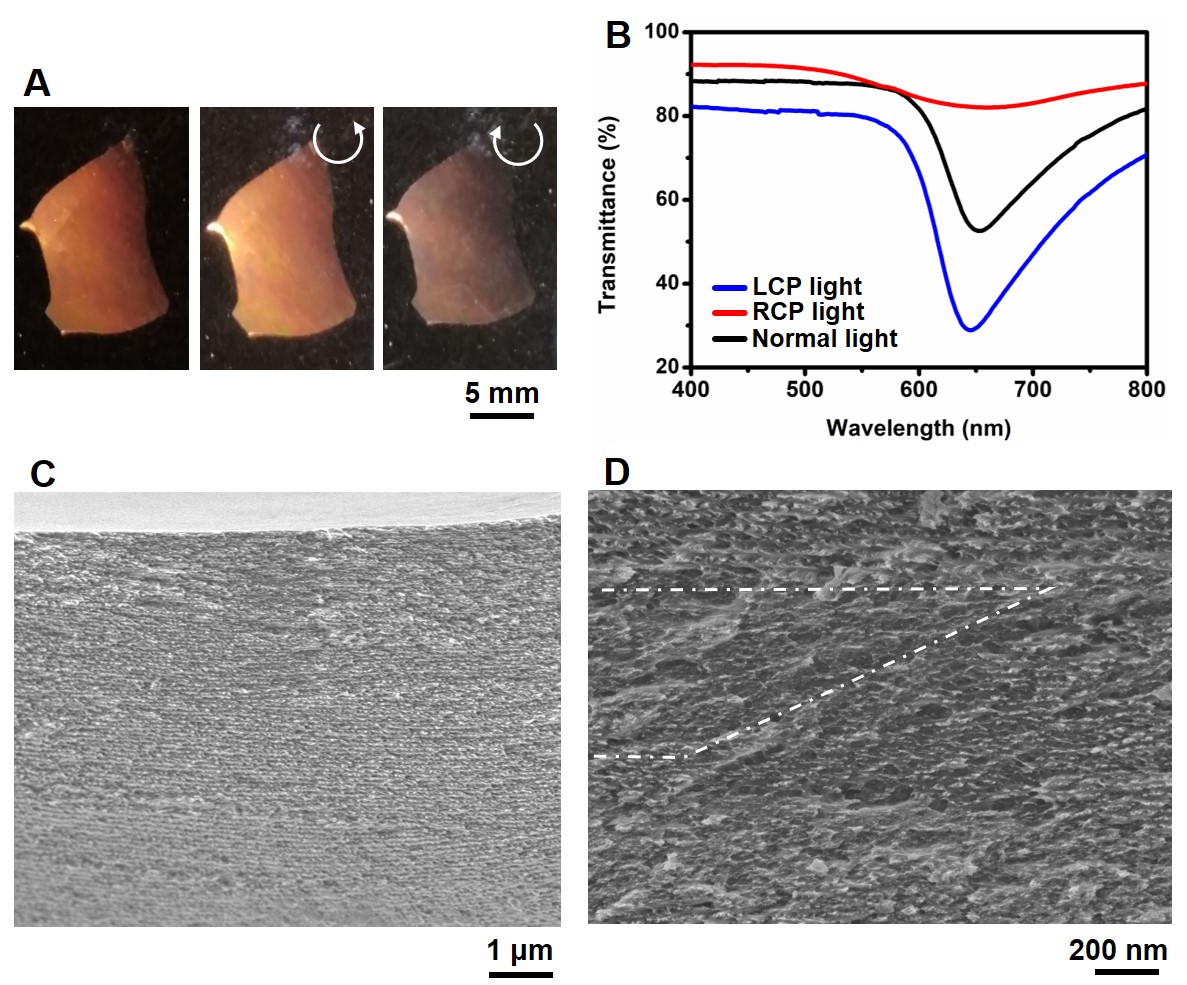
**

**Supplementary Figure S2.** (A) Photographs of CNC-0 viewed under normal white light (left), through the LCP filter (middle) and the RCP filter (right), (B) transmission spectra of CNC-0 probed by normal white light (solid black line), through the LCP (solid blue line) or RCP filter (solid red line), (C-D) SEM images show a chiral nematic architecture (C), where the nematic-like layers (dotted white circles) are embedded between left-handed PBG layers (D).


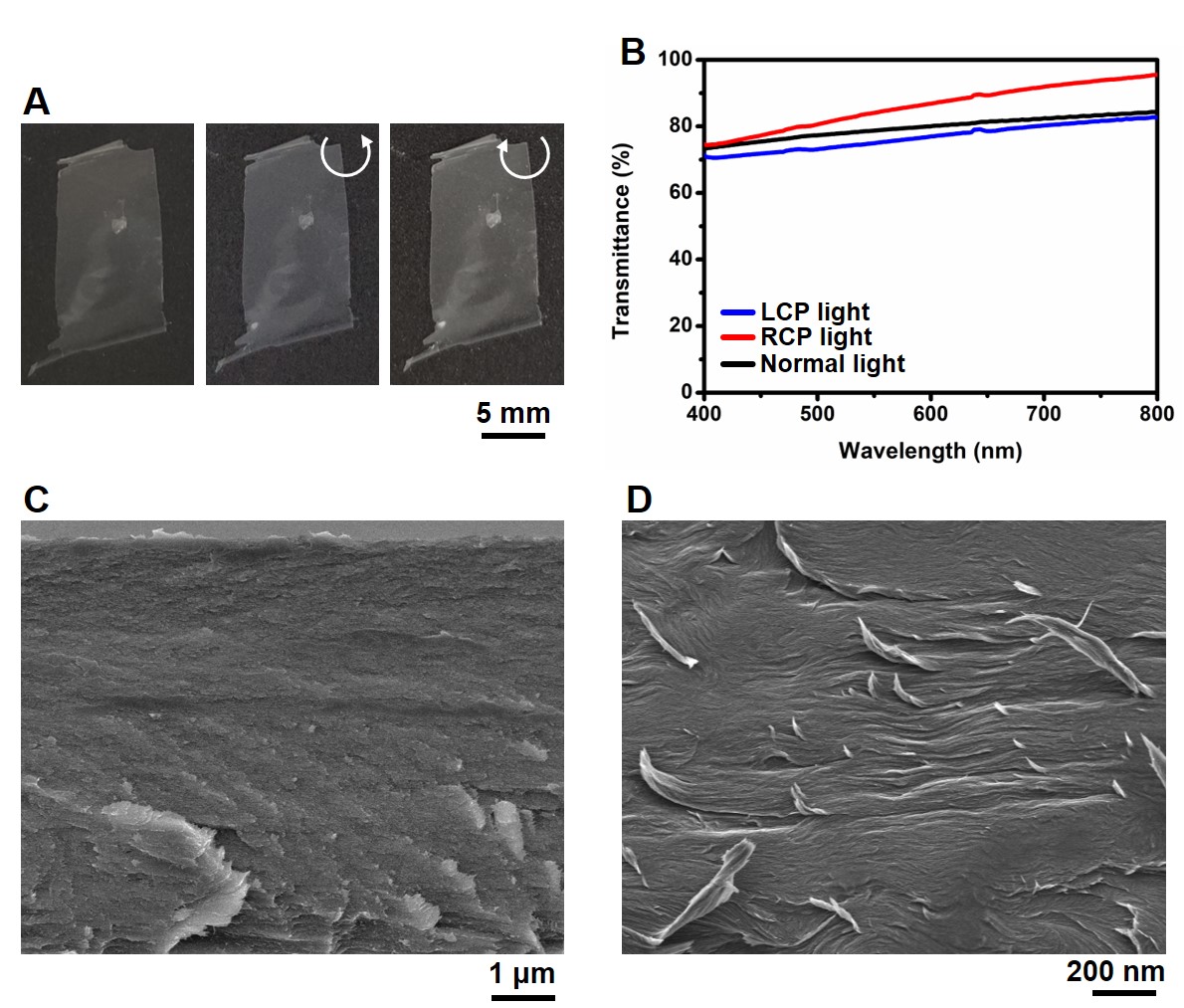


**Supplementary Figure S3.** (A) Photographs of CNC-90 viewed under normal white light (left), through the LCP filter (middle) and the RCP filter (right), (B) transmission spectra of CNC-90 probed by normal white light (solid black line), through the LCP (solid blue line) or RCP filter (solid red line), (C-D) SEM images show unidirectional alignment of CNC (C) along film lateral direction (D).
